# Host Specificity and Temporal and Seasonal Shifts in Host Preference of a Web-Spider Parasitoid *Zatypota percontatoria*

**DOI:** 10.1673/031.011.10101

**Published:** 2011-08-15

**Authors:** Stanislav Korenko, Veronika Michalková, Kees Zwakhals, Stano Pekár

**Affiliations:** ^1^ Department of Agroecology and Biometeorology, Faculty of Agrobiology, Food and Natural Resources, Czech University of Life Sciences Prague, Kamýcká 129, 165 21 Prague 6, Suchdol, Czech Republic; ^2^ Department of Botany and Zoology, Masaryk University, Faculty of Science, Kotlářská 2, 611 37 Brno, Czech Republic; ^3^ Institute of Zoology, Slovak Academy of Sciences, Dúbravská cesta 9, 845 06 Bratislava, Slovakia; ^4^ Dr. Dreeslaan 204, NL-4241 CM Arkel, Netherlands

**Keywords:** foraging strategy, host-parasitoid interaction, *Neottiura bimaculata*, *Theridion varians*

## Abstract

Current knowledge about polysphinctine parasite wasps' interactions with their spider hosts is very fragmented and incomplete. This study presents the host specificity of *Zatypota percontatoria* (Müller) (Hymenoptera: Ichneumonidae) and its adaptation to varying host availability. Two years of field observations show that *Z. percontatoria* is a stenophagous parasitoid that parasitizes only five closely related web-building spiders of the family Theridiidae (Araneae). Within the Theridiidae it attacks only species belonging to a small group of species, here called the “*Theridion*” group. These hosts have a similar biology, but are available at different levels of abundance and at different sizes over the season. Laboratory experiments showed that this wasp species ignores linyphiid, araneid or dictynid spiders and accepts only theridiid spiders of the “*Theridion*” group. In the field study, wasp females preferred older juvenile and sub-adult female spider instars with intermediate body size. Only 5% of the parasitized spiders were males. Parasitism in the natural population of theridiid spiders was on average 1.3%. Parasitism was most frequent on two species, *Theridion varians* Hahn in 2007 and *Neottiura bimaculata* Linnaeus in 2008. The parasitization rate was positively correlated with spider abundance. The wasp responded adaptively to seasonal changes in host abundance and host body size and shifted host preference according to the availability of suitable hosts during, as well as between, seasons. In spring and summer the highest percentage of parasitism was on *T. varians* and in autumn it was on *N. bimaculata.*

## Introduction

Foraging is one of the central issues in ecology as it shapes the interactions between predators and their prey. Foraging of a parasitoid includes attack on a single free-living host and oviposition of an egg that develops only on the host tissue leading to complete host consumption. Due to this restriction, parasitoids have evolved adaptive foraging and host-selection strategies that enable them to survive in situations when host sources are restricted (Godfray and Shimada 1999).

Two different parasitoid developmental strategies exist: idiobiosis and koinobiosis. Idiobiont species develop on a paralyzed and non-growing host (Askew and Shaw 1986), hence their host resource is static in terms of size during their development. Parasitoid size is thus a function of host species size at the time of attack. By contrast, koinobiont species attack hosts that continue to feed and grow (Askew and Shaw 1986). The amount of resources exploited by such parasitoid larvae for growth and development is not fixed; their development depends upon host feeding rate and growth (Godfray 1994).

Hymenopteran parasitoids show a great variety of host specializations. The host range of specialist parasitoids, assessed at the taxonomic level, is often very narrow, whereas generalists exploit a broad range of hosts (e.g., Shaw 1994; Shaw and Aeschlimann 1994). They tend to attack a group of host species that share a similar set of characteristics, either ecological or phylogenetic. When encounters with one host species become infrequent, generalist parasitoids expand their host selection criterion to include other (even less profitable) host species (Henry et al. 2009). As a result, generalist parasitoids can have multiple generations per season (Pyke et al. 1977).

Koinobionts are generally more host-specific than idiobionts (Althoff 2003). In strictly specialized parasitoids, their life history is tightly coupled with that of their host species. Such parasitoids occur only in the habitat of their host and synchronize their development with them (Barrantes et al. 2008). Examples of rather specialized parasitoids include polysphinctine ichneumonids that attack spiders (e.g. Fitton et al. 1987).

Host size or developmental stage vary during the season. Therefore, choosing suitable hosts is fundamental to the parasitoid—host relationship. Some parasitoids, for example, attack hosts only at a certain developmental stage or size (Henry et al. 2006). Henry et al. (2009) demonstrated that smaller parasitoids receive the greatest benefits from selecting small, but less defended and abundant hosts. Hosts, such as web spiders, are habile hunters that can easily overcome the handling capabilities of smaller parasitoids (Gentry and Dyer 2002; Henry et al. 2006). The host's ability to physically defend itself typically increases with host age and size. Female parasitoids must make a choice between increased handling time and risking injury in attempting to oviposit on larger dangerous hosts (Gerling et al. 1990).

The koinobiont ectoparasitoid wasp, *Zatypota percontatoria* (Müller) (Hymenoptera: Ichneumonidae), belongs to polysphinctine pimplines, which exhibit a unique trait within the Ichneumonidae in terms of development (Fitton et al. 1987). A wasp larva develops externally on an active spider and the spider host is killed and consumed shortly before the ichneumonid's pupation. Biologically and morphologically, polysphinctines belong to the most specialized Pimplinae (Fitton et al. 1987). Overall, the trophic relationship between polysphinctines and spiders is clearly highly specialized, and *a priori* it is expected that the host ranges of these species are narrow (Shaw 1994). Our knowledge about this host — parasitoid system still remains very fragmented and incomplete.

The few available records about the *Zatypota* species show that it attacks spiders from several different families (Aubert 1969; Shaw 1994; Gauld and Dubois 2006). Shaw (1994) summarized host records of *Z. percontatoria* and concluded that this species attacks spiders from several web-building families, namely Araneidae, Dictynidae, Tetragnathidae and Theridiidae. However, Gauld and Dubois (2006) presumed a misidentification of the reared wasp in several cases and questioned all hosts other than theridiid spiders. Thus, verified records of hosts of *Z. percontatoria* include only a few spider species from the family Theridiidae: *Phylloneta impressa* (L. Koch), *Platnickina tincta* (Walckenaer), *Theridion varians* Hahn, *T. simile* Koch and *T. melanurum* Hahn (Aubert 1969; Fitton et al. 1987; Gauld and Dubois 2006).

The aim of this study was to investigate the foraging strategy of *Z. percontatoria* in terms of host specificity at a taxonomic, developmental and sexual level.

## Materials and Methods

### Field study

A population of *Z. percontatoria* was investigated in an apple orchard situated in Brno, Czech Republic (49° 09′ 37″ N, 16° 33′ 35″ E). The orchard consists of two tree varieties, Golden Delicious and Champion, both between 27 and 30 years old. The orchard was under an integrated pest management regime during our study.

The percentage of parasitism (defined as the total number of cases of parasitized spiders in the population at a given time, divided by the number of individuals in the population) was investigated during 2007 and 2008, by collecting potential and parasitized hosts. The hosts, arboreal spiders, were collected by beating apple tree branches. This was done from spring (April) to late autumn (October) each year. Spiders were collected in a square shaped beating net (1m^2^ area) placed beneath the tree crown. A single sampling consisted of beating the branches of 25 trees. The sampling was performed on 12 days in April, May, August, September, and October in 2007 and on 10 days in April, May, June, July, August, and September in 2008. On each investigated day three samples were taken. All spiders were fixed in 70% alcohol and identified to species/genus level using Heimer and Nentwig (1991), Roberts (1995) and Pekár (1999). The spider nomenclature is according to Platnick (2009).

During both years, 11 theridiid spider species were identified to species level based upon adult specimens. The majority of these belonged to the “*Theridion*” group. In the terms of this study the “*Theridion*” group includes the following closely related species with a similar biology and web architecture (see, e.g. Heimer and Nentwig 1991): *Theridion varians* Hahn, *T. pinastri* L. Koch, *Phylloneta impressa* Linnaeus, *Neottiura bimaculata* (Linnaeus), *Platnickina tincta* (Walckenaer), *Heterotheridion nigrovariegatum* (Simon) and *Paidiscura pallens* (Blackwall). The remaining species are *Dipoena melanogaster* C. L. Koch, *Enoplognatha latimana* Hippa and Oksala, *E. ovata* (Clerck) and *Parasteatoda lunata* (Clerck). The body size (prosoma length), developmental stage (juvenile or adult), and sex of each parasitized spider were determined with an Olympus stereomicroscope SZ 40.

The sex ratio of both unparasitized (N = 204) and parasitized (N = 75) “*Theridion*” spiders was estimated from individuals of sub-adult and adult growth stage. The sex of spiders could be recognized only at later juvenile and sub-adult stages, i.e. when their prosoma was larger than 0.7 mm. At these stages, males were recognized by swollen pedipalps and females by a swollen and dark area of the epigyne. The length of the prosoma is a commonly used parameter for body size because unlike the length of the abdomen it changes only during molting (Schaefer 1987).

### Wasp rearing

Living parasitized spiders were collected in the same periods but in a different part of the orchard. Parasitized spiders (N = 138) were collected by means of branch beating. Hosts with parasitoids were placed singly in cylindrical containers (diameter 35 mm, height 40 mm) with a layer of plaster of Paris at the bottom. The plaster was moistened at three-day intervals. Spiders were kept at room temperature 22 ± 3.5° C under a natural L:D regime and fed with a surplus of *Drosophila melanogaster* Meigen (Diptera: Drosophilidae). The spiders were reared until the wasps' emergence. Spider hosts were identified and the results were included into host-specificity frequencies. Hatched wasps were identified using Fitton et al. (1988) and Zwakhals (2006). The nomenclature of the polysphinctines follows Fitton et al. (1988) and Yu and Horstmann (1997). Some of these wasps were used in the experiments described below.

### Laboratory experiments

The host preference of *Z. percontatoria* was investigated under laboratory conditions in two experiments. In the first one, the preference for hosts of four spider genera belonging to four families was tested; in the second one, the preference for spider species of one family was tested. A similar experimental set-up was used in both experiments. After hatching, the virgin female wasps were placed singly in glass vials (height 85 mm, diameter 55 mm), with a cotton ball soaked in 20% aqueous honey solution at the bottom. Three days later three spiders (potential hosts) were released into each vial simultaneously and acceptance of a particular spider host was recorded within 48 hours. Arrhenotokous parthenogenesis of virgin females seems to be common in *Z. percontatoria* (Korenko, unpublished).

In the first experiment with 18 wasp females, web-building spiders, specifically *Araniella* sp. (Araneidae, N = 12), *Meioneta* sp. (Linyphiidae, N = 11), *Dictyna* sp. (Dictynidae, N = 13), and *Theridion* spp. (Theridiidae, N = 18) collected in the orchard, were offered as potential hosts. The prosoma length of these spiders was between 0.6 and 1 mm. All spiders were juvenile and did not build a complete web in such a short time. If the spider built a dense web, it was destroyed with a stick in order to prevent the female wasp from becoming caught in the web.

In the second experiment with 30 wasp females, six species of the family Theridiidae were offered. The following species were used: *N. bimaculata* (N = 17), *P. impressa* (N = 11), *T. varians* (N = 30), “*Theridion*” (N = 15), *Dipoena melanogaster* (N = 9) and *Enoplogntha* sp. (N = 8). All spiders were juvenile with a prosoma length between 0.6 and 1 mm.

### Data analysis of the laboratory experiments

Data were analyzed using various methods within the R environment (R Development Core Team 2009). The Chi-square “goodness of fit” test was used to compare frequencies of abundance of potential hosts with the rate of parasitized hosts. The Proportion test was used to compare proportions of each sex among available and parasitized hosts. ANOVA was used to compare host sizes during each season, as the response variable was continuous and residuals were homoscedastic. The results of laboratory experiments were analyzed using two different methods. As in both experiments, observations of the frequency of parasitization were not independent due to blocked design, therefore methods that can handle correlated data were used. The first experiment included only 18 vials (blocks); thus, the Generalized Linear Model with a quasibinomial setting (GLM) was used to correct for over-dispersion due to the correlated response within vials (Pekár and Brabec 2009). The second experiment included 30 vials (blocks); therefore, Generalized Estimating Equations with binomial error structure (GEE) were used (Hardin and Hilbe 2003). Within this model an exchangeable association structure was used.

## Results

### Host specificity from the collected spiders

Of 9,314 spider specimens collected in the field, the potential spider hosts, i.e. the webbuilding spiders, were represented by five families amounting to 85.7% of all collected spiders. These were Theridiidae (92%, N = 7,353), Araneidae (6.2%), Dictynidae (0.5%), Tetragnathidae (0.6%) and Linyphiidae (0.6%). The parasitoid, however, attacked only Theridiidae (100%, N = 105).

**Figure 1.**
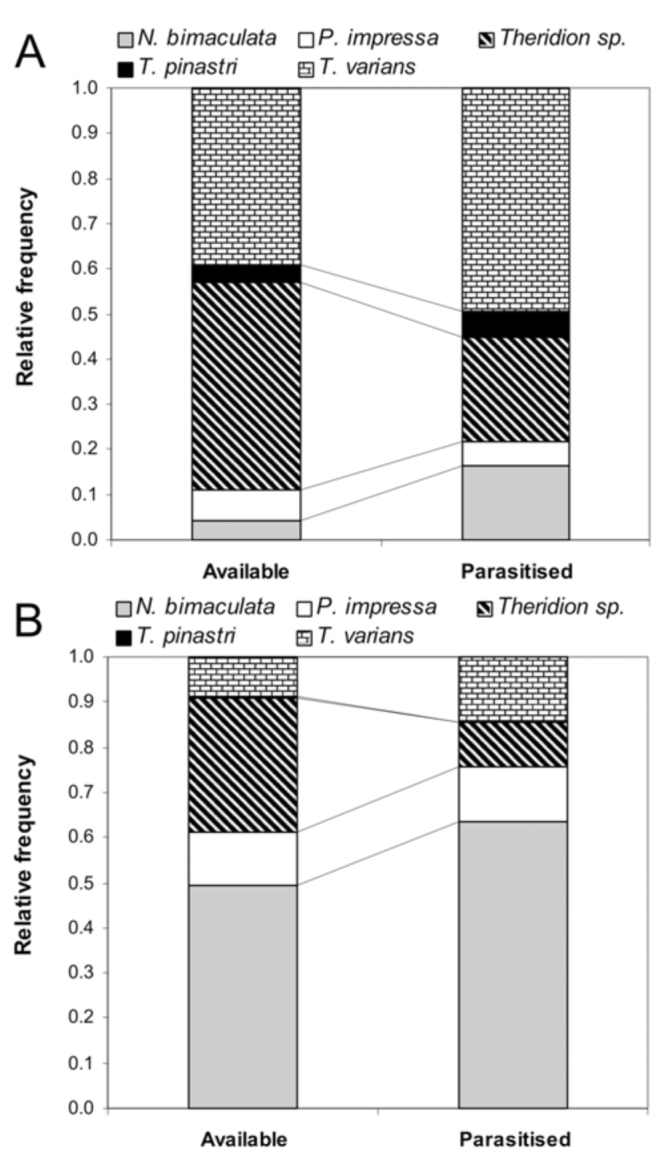
Comparison of relative frequency of available and parasitized “*Theridion*” spider hosts in the field in 2007 (A) and 2008 (B). High quality figures are available online.

Using all data from both the samples from the parasitism rate study (N = 105) and the rearing of wasps (N = 138), it was shown that parasitized theridiid spiders belonged mainly to *Theridion varians* (43%, N = 104), *Neottiura bimaculata* (24.4%), *Phylloneta impressa* (6.6%), *T. pinastri* (4.5%) and unidentified juveniles of the “*Theridion*” group (21.5%). The frequency of attacked theridiid hosts did not correspond to their availability both in 2007 (Goodness of fit, χ^2^_4_ = 1674, *p* < 0.0001) and in 2008 (Goodness of fit, χ^2^_4_ = 1137, *p* < 0.0001), (Figure 1).

**Figure 2.**
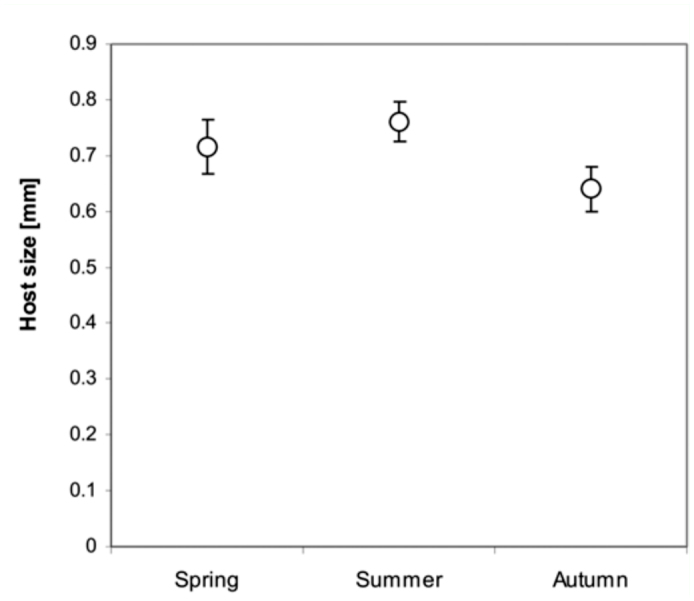
Comparison of the size (prosoma length) of available juvenile “*Theridion*” hosts during season. Points are means; uncertainty bars represent 95% confidence intervals. High quality figures are available online.

The vast majority of the parasitized spiders (89.5%) were at the juvenile stage (from N = 105). Adults, which comprised 10.5% of all parasitized hosts, were parasitized merely in August 2007, and these were exclusively females of *T. varians.* The mean size of the prosoma of the parasitized host was 0.71 mm (N = 105, SE = 0.013) and changed somewhat during the season (Figure 2).

The natural sex ratio of subadult and adult “*Theridion*” spiders was biased in favor of females, as only 25% (from N = 204) of spiders were males. The sex ratio of parasitized “*Theridion*” spiders was significantly skewed in favor of females, as only 5% (from N = 75) of spiders were males (Proportion test, χ^2^_4_ = 12.2, *p* = 0.0005).

### Ovopositioning results in the laboratory

**Figure 3.**
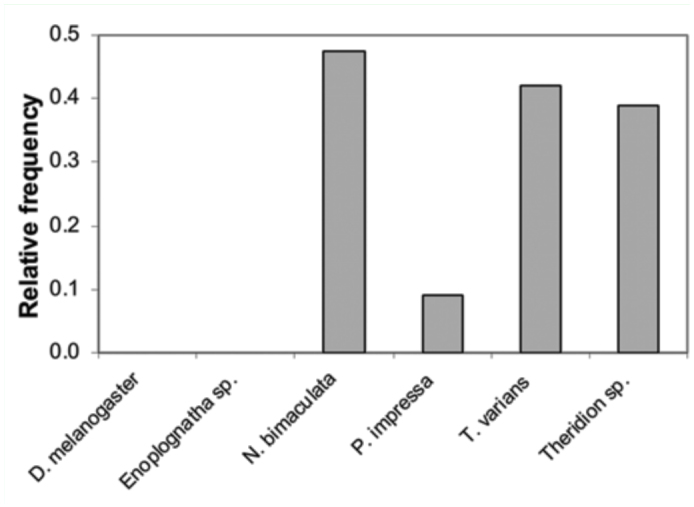
Relative frequencies of six taxa parasitized by wasps in laboratory experiments. High quality figures are available online.

Under laboratory conditions, *Z. percontatoria* attacked exclusively spiders of the family Theridiidae and ignored linyphiid, araneid or dictynid spiders (GLM, *F*3,32 = 19.1, *p* = 0.004). When six taxa of the family Theridiidae (N = 30) were offered, parasitism differed significantly among them (GEE, χ^2^_5_ = 87.2, *p* < 0.0001). *N. bimaculata, T. varians* and “*Theridion*” were parasitized with similarly high frequency, *P. impressa* was parasitized with a low frequency, and *D. melanogaster* and *Enoplognatha* sp. were not parasitized at all (Figure 3).

### Host shifts

In 2007 the rate of parasitism by *Z. percontatoria* wasps on theridiid spiders was 1.74% (from N = 4,814). In 2008 the rate halved to 0.83% (from N = 2,539). Comparison of the host spectrum between the two seasons, 2007 and 2008, revealed a shift in host preference. While in 2007 the highest portion of parasitized hosts was represented by *T. varians*, in 2008 it was represented by *N. bimaculata* (Figures 1A, B). In 2007 (April – August) *T. varians* was the most abundant species with a mean abundance of 40.1 specimens (N = 7, SD = 23.5). This species was also the most parasitized (49.5%) spider host (Figure 1A). In 2008, the abundance of *T. varians* decreased to 12.9 (N = 8, SD = 13.8), and its parasitized portion decreased to 14.6%. *Neottiura bimaculata* had a lower abundance in 2007, specifically 4.3 (N = 7, SD = 4.4) spiders and a parasitized portion 16.3%. In 2008, it was the most abundant species with 20.9 specimens (N = 8, SD = 6.5) and a higher portion of parasitism (63.4%) (Figure 1B). The abundance of *P. impressa* was similar in both years, i.e. 7 specimens per day (N = 7, SD = 9.8) in 2007 and 5.4 specimens (N = 8, SD = 4.84) in 2008. Its portion of parasitism increased from 5.4 to 12.2% between years.

**Figure 4.**
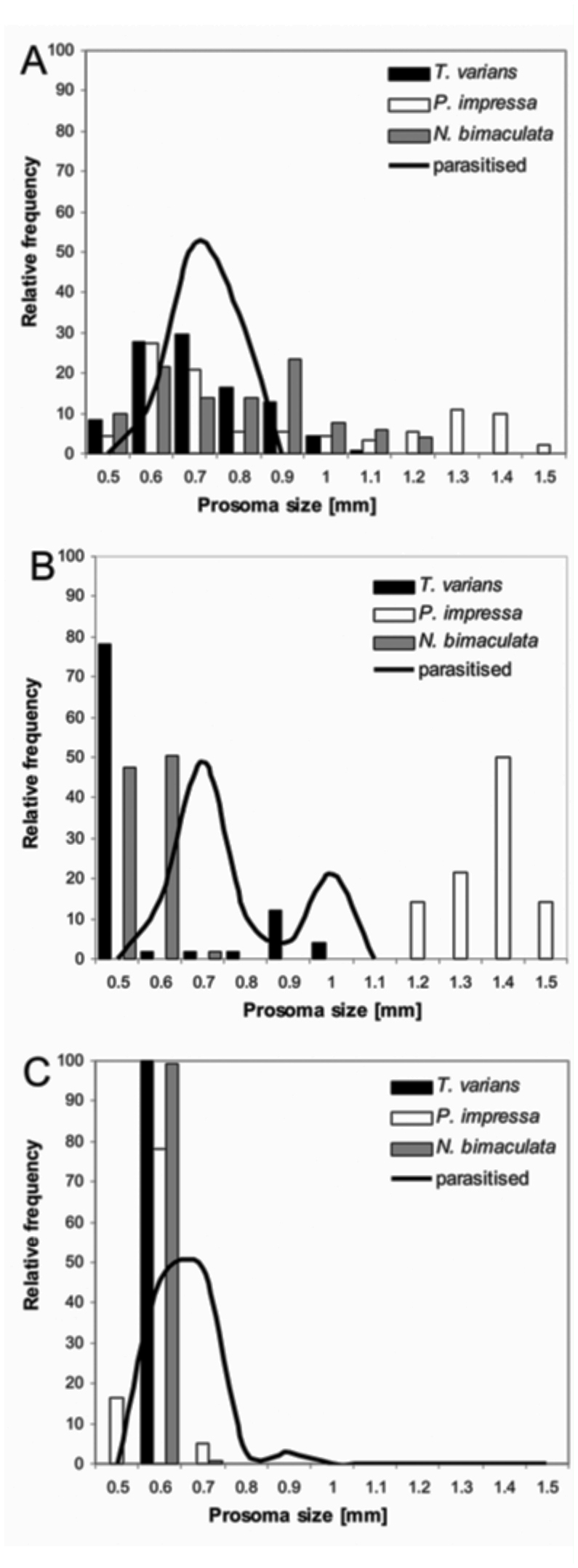
Relative frequencies of prosoma sizes of three major available host species (bars) and parasitized individuals (line) in spring (A), in summer (B) and in autumn (C). Bars were generated from spider relative abundance in the particular prosoma size (for each species separately). Lines were generated from all parasitized spiders in the particular prosoma size collected in the field in the particular season. High quality figures are available online.

The body size of parasitized hosts changed significantly according to the seasons (ANOVA, F_2,102_ = 10.4, P < 0.0001): it was smallest in autumn and largest in summer (Figure 2). The frequency of occurrence of the three main hosts, *T. varians, N. bimaculata* and *P. impressa,* at a size preferred by the parasitoid, also changed with the seasons. In spring, all three host species were juvenile and thus occurred at high frequencies at the preferred body size (Figure 4A). In spring (of both years) the highest parasitized portion was in *T. varians* (43.5%, N = 10), followed by *N. bimaculata* (26%), the “*Theridion*” group (15.5%), *P. impressa* (13%) and *T. pinastri* (2%). In summer, spiders with a suitable body size were rare, as the majority of them had reached adulthood. The prosoma size of adult *T. varians*, however, falls within the preferred host range and adult females were thus accepted as host (Figure 4B). This was not the case in *P. impressa,* whose adult prosoma length was far larger than 1.1 mm. Neither the first instar spiderlings (prosoma size < 0.5 mm) of *T. varians* or *N. bimaculata* that hatched from egg sacs were accepted as hosts (field data). In summer, the highest parasitized portion was observed on *T. varians* (57%, N = 26), followed by indeterminable spiders at early instars (prosoma > 0.6 mm) of the “*Theridion*” group (26%), *N. bimaculata* (8.7%), and *P. impressa* (6%) and *T. pinastri* (2.3%). In autumn, spiderlings (prosoma size > 0.6 mm) of all three species were accepted by the wasp (Figure 4C). The parasitized portion was highest on *N. bimaculata* (37%, N = 13), followed by *T. varians* (28.6%), indeterminable spiders at early instars of “*Theridion*” group (25.7%), *P. impressa* (5.7%) and *T. pinastri* (3%).

## Discussion

Both field and laboratory data show a strong preference of *Z. percontatoria* for webbuilding spiders of the family Theridiidae. It is known that host identification may be based on visual and chemical cues and the attacking behavior may be dependent on the invasion of the host web (Fellowes et al. 2005). The limitations of the performed laboratory experiments (i.e. with only virgin females enclosed in a small container and spiders deprived of their webs) could have influenced the searching and attacking behavior of the wasp. We assume that wasp females, which naturally mate in the field, may express different host acceptance behaviors. If female wasps allocate male offspring to smaller hosts and female offspring to larger hosts (e.g. Takasuka et al. 2009), then patterns of host preference for virgin females might differ from those of mated females.

Although laboratory experiments have limitations, they clearly support field data. The same spider species were accepted by wasps in the laboratory as in the field. Thus, as predicted by Gauld and Dubois (2006), previous records of hosts from other families, namely Agelenidae, Araneidae, Dictynidae and Tetragnathidae, cannot be supported. *Zatypota percontatoria* clearly attacks spiders building 3-dimensional webs with similar biology and web architecture. This study confirmed three hosts published previously (Aubert 1969; Fitton et al. 1987; Gauld and Dubois 2006) and revealed two new hosts: *N. bimaculata* and *T. pinastri.*

The results of this study provide data only for a single population. To identify the entire host spectrum of *Z. percontatoria*, studies in other places of its distribution area should be undertaken. This species has a Holartic distribution, but most of the records come from Europe, where about 30 spider species of the family Theridiidae belong to the potential pool of hosts.

### Host size/stage preference

Within the “*Theridion*” group we found *Z.* *percontatoria* to select only hosts of a certain size range. Populations of spider hosts are generally characterized by a high degree of body size variation (Henry et al. 2009). Large hosts provide more resources and, therefore, produce larger parasitoids, which is a proxy mechanism positively correlated with parasitoid fitness (Sequeira and Mackauer 1994; Takasuka et al. 2009). Parasitoids can distinguish between high- and low-quality hosts and preferentially oviposit on higher quality ones (Godfray 1994). Fincke et al. (1990) observed an intermediate-size preference for juvenile female spiders by *Hymenoepimecis* sp. in Panama. This is because the developmental stage of a host becomes far more important for larva survival than the actual size of the host at the moment of parasitization (Henry et al. 2006).

*Zatypota percontatoria* is a medium-sized parasitoid and it attacks medium-sized spiders corresponding to older juvenile and sub-adult spider instars. It does not accept hosts that are either too small (early instars) or too large (adult specimens). Tiny spiders are probably not accepted because the larva would be too great a burden for the spider, likely decreasing its foraging efficiency, which would negatively affect the fitness of the parasitoid. *Zatypota percontatoria* females significantly preferred female juvenile spiders as host. Generally, female spiders are larger, can be more abundant and have higher longevity (e.g. Schaefer 1987); therefore, they are a more profitable source of food for larvae than males. The selection of female sex in spider hosts is profitable due to divergent ontogenetic developmental trajectories. As females are larger than males at the adult stage, they have a higher foraging rate (Givens 1978), pass through more instars and attain a larger body size (Vollrath 1987). We can assume that adult males are avoided due to their short life expectancy, while large adult females are presumably avoided because of their stronger defenses against an ovipositing female wasp. It is, however, not known how wasps recognize the sex of immature spiders.

### Host shifts

*Zatypota percontatoria* adaptively changed hosts according to their size, which was changing during the season. The two most preferred hosts, *T. varians* and *N. bimaculata,* attain an acceptable body size even at maturity, but the latter species was not parasitized at the adult stage. This is probably because adult female specimens of *N. bimaculata* are hidden while guarding their eggsac underneath the leaves of an herb layer (Korenko, unpublished). Thus adult females of *T. varians* were the only acceptable hosts of the preferred size in the middle of a season, presumably because they build webs at a higher vegetation stratum.

Species of the “*Theridion*” group are among the most abundant spiders in crowns of fruit trees (Pekár and Kocourek 2004). In central Europe, three species, *T. varians, N. bimaculata* and *P. impressa* are the most abundant 3-D web weavers, but they differ in phenology and body size during the season. *Zatypota percontatoria* responds adaptively to changes in abundance, body size and age of the three hosts. Spider hosts may become unavailable, for example due to reproduction when males die soon after mating, or due to large body size, which is not accepted by the wasp. This adaptive behavior is an effective means of maintaining a stable population dynamic throughout the season.

We also observed shifts in host preference between years. In the first year, 2007, *T. varians* was the dominant host, while in the second year, 2008, the parasitoid shifted to *N. bimaculata.* Parasitoids may influence parasitoid—host dynamics (Mangel and Roitberg 1992), which can further produce cascading effects on populations that shape the structure of the entire arthropod community (Petchey et al. 2008). Theridiid spiders are polyphagous predators, very abundant in agrobiocenoses and thus important for the natural control of pests (Bogya 1999). The rate of parasitism on theridiid hosts by *Z. percontatoria* was relatively low in both years. This complements observations on other related species: 1.4% in *Zatypota petronae* Gauld parasitized on *Theridion evexum* Keyserling (Barrantes et al. 2008) and 5% in *Zatypota albicoxa* (Walker) parasitized on *Parasteatoda tepidariorum* (C.L.Koch) (Tanaka 2007). In the study orchard, the frequency of parasitism did not exceed 6% throughout the season, except for the summer of 2007, when parasitism rate was 33% (sample in August). This high increase in parasitism rate was caused by a low abundance of acceptable theridiid hosts. Adult females of *T. varians* were frequently parasitized in the summer of the first year of the investigation (50% of all *T. varians* specimens). In general, such a situation could lead to a dramatic decline in the population of this species in the following year. The impact of parasitism on the host population seems to be most disastrous when adult females are attacked. The high rate of parasitism on adult female hosts was observed by Eberhard (2000) and Gonzaga and Sobezak (2007), but they did not investigate how parasitism rate influences host population dynamics.

This study confirms that *Z. percontatoria* is a stenophagous parasitoid attacking a group of ecologically similar and taxonomically related spider hosts. Such narrow host specialization is advantageous because it requires similar attack and handling behavior. Not being strictly monophagous allows shifts between hosts when their availability changes during and between seasons.
